# Quantifying the contribution of microbial immigration in engineered water systems

**DOI:** 10.1186/s40168-019-0760-0

**Published:** 2019-11-06

**Authors:** Ran Mei, Wen-Tso Liu

**Affiliations:** Department of Civil and Environmental Engineering, University of Illinois at Urbana-Champaign, Urbana, IL USA

**Keywords:** Microbial immigration, Mass balance, Engineered water systems, Microbiome

## Abstract

Immigration is a process that can influence the assembly of microbial communities in natural and engineered environments. However, it remains challenging to quantitatively evaluate the contribution of this process to the microbial diversity and function in the receiving ecosystems. Currently used methods, i.e.*,* counting shared microbial species, microbial source tracking, and neutral community model, rely on abundance profile to reveal the extent of overlapping between the upstream and downstream communities. Thus, they cannot suggest the quantitative contribution of immigrants to the downstream community function because activities of individual immigrants are not considered after entering the receiving environment. This limitation can be overcome by using an approach that couples a mass balance model with high-throughput DNA sequencing, i.e.*,* ecogenomics-based mass balance. It calculates the net growth rate of individual microbial immigrants and partitions the entire community into active populations that contribute to the community function and inactive ones that carry minimal function. Linking activities of immigrants to their abundance further provides quantification of the contribution from an upstream environment to the downstream community. Considering only active populations can improve the accuracy of identifying key environmental parameters dictating process performance using methods such as machine learning.

## Introduction

Microbial communities play essential roles in biogeochemical cycles in natural and engineered ecosystems [1]. To study how different microorganisms assemble into a community and contribute to the function of an ecosystem, various mechanisms including the niche and neutral theories have been developed [2]. In the neutral theory of biodiversity and biogeography, immigration is one of the key stochastic processes that change the community assemblage together with death and birth [3]. This process, sometimes referred to as migration [4], is originally used in macroecology to estimate the rate of new bird species entering a remote island from the nearest land mass, i.e., the chance of immigration, which plays a pivotal role in the equilibrium of island fauna’s diversity [5]. As the definition of immigration can vary considerably [6], this review adopts the one stated by Bell [4], and defines immigration as the process of a microbial individual being added to a local community from the species pool of the metacommunity, which consists of a set of local communities that are physically linked by immigration and can exchange colonists of multiple species [7]. A similar term often used is dispersal [8–10]. While dispersal and immigration may slightly differ in specific context and one may even include the other [6], there is still no consensus on the difference [10–13]. Therefore, we do not attempt to discuss the differences between immigration and dispersal here. We further consider microorganisms that arrive at the local communities all as immigrants, regardless of how they arrive (e.g., facilitated by cell motility or flow of water or air) and how they contribute to the local community after arrival.

**Fig. 1:**
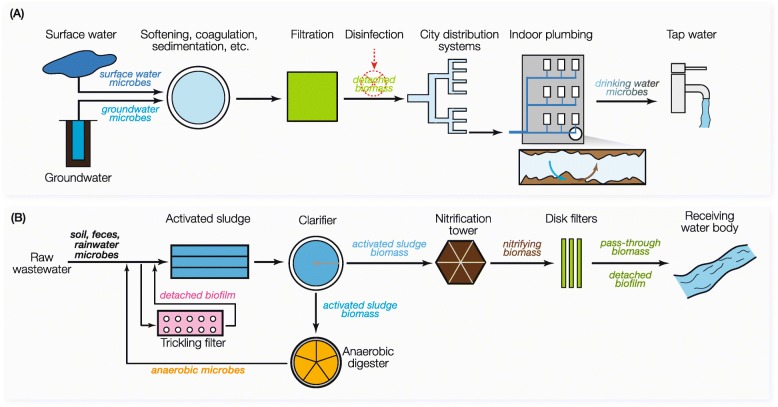
Illustration of potential microbial immigration in **a** drinking water production and distribution system, and **b** wastewater treatment plants

In natural microbial ecosystems, immigration from an upstream community to a downstream community can be exemplified by microbial immigration from Africa dust to European aquatic environments [14], from running waters to forest lakes [15], and from river water to estuarine and offshore environments [16]. Microbial immigration also widely occurs in engineered environments, and this movement often occurs in a more controlled and directed manner than in natural environments. In drinking water production systems (Fig. 1a), microorganisms in the source water, e.g.*,* groundwater and surface water, can serve as inocula to seed the communities along the treatment process [17]. Next, microbes growing on the treatment units, e.g.*,* surfaces of reactor walls or filtration media, can be released to subsequent processes [18] and then to the distribution system depending on whether disinfection is practiced [19] or not [20]. In the distribution network and indoor plumbing, bidirectional immigration can occur between the water phase and the biofilms on the inner pipe surface [21], which can be further elevated through water stagnation [22]. A wastewater treatment plant (WWTP) is another good example of microbial immigration (Fig. 1b), because different bioreactors are physically connected, and the flux of biomass can be higher than those in natural systems. In WWTPs, microbes present in raw sewer [23] or nitrifying trickling filter [24] were reported to have impact on the downstream activated sludge communities. The activated sludge biomass can also act as a source of immigration to the downstream anaerobic digester microbiome [25]. The WWTP effluent can impact the receiving water body community [26], elevating the abundance of human gut-related microbes and antibiotic resistance genes [27].

While microbial immigration is frequently reported in engineered water systems, it remains challenging to quantitatively address “to what extent immigration contributes to the assembly and function of the downstream community?” In this article, we focus on methodology quantifying microbial immigration by first reviewing methods that are currently used and identifying their limitations. Then, an approach that calculates the net growth rates of individual microbial immigrants in the downstream community is reviewed. It couples a mass balance model with high-throughput DNA sequencing to partition microbial assembly into active populations that contribute to community function and inactive ones that carry minimal microbial function. Its potential use together with machine learning to identify key environmental parameters affecting the microbial ecosystem’s function is discussed.

## Methods commonly used to evaluate immigration impact

**Fig. 2:**
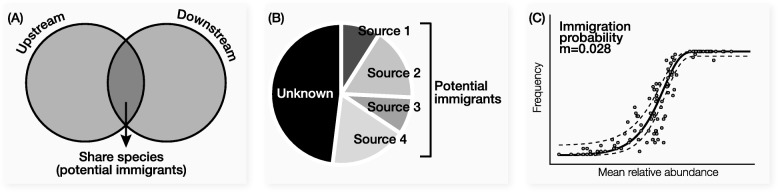
Current methods to quantify immigration impact. **a** Counting shared species with Venn diagram, **b** microbial source tracking, and **c** Sloan’s neutral model

Figure 2 illustrates three methods commonly used to evaluate immigration impact in microbial ecosystems. The first approach simply counts shared species between the upstream and downstream communities, as visualized by a Venn diagram (Fig. 2a). For example, McLellan et al. reported that untreated sewage communities shared more species with surface water communities than with human fecal sources, indicating surface water such as rainwater and storm water can modulate sewage microbial community composition [28]. Lee et al. compared microbial communities between influent wastewater and the downstream activated sludge in four full-scale WWTPs and reported that shared species accounted for 12.2%, 7.5%, 15.2%, and 7.6% of total sequences in activated sludge, respectively [23]. As the authors stated, these numbers only implied “contribution from influent wastewater communities to some extent”, while the exact contribution to the function of each activated sludge system cannot be quantified. This uncertainty is due to the fact that the microbial activity of immigrants cannot be determined. Shared species can be generalists that function in both upstream and downstream environments or be detectable but remain dormant in activated sludge due to their high abundance in the influent and low adaptability (i.e., low activity) to the new environmental conditions.

The second approach is microbial source tracking that estimates the proportion of taxa in the downstream or sink community coming from multiple upstream or source environments [29] (Fig. 2b). The basic rationale is that more abundant taxa in the source have higher probabilities to be observed in the sink, which represents the contribution of each source. This method has been applied to study a sink environment that receives immigration from multiple sources, such as residential kitchen microbiome subject to source microbiota of the human palm skin, produces, and faucet water [30] and public restroom microbiome subject to source microbiota of the soil, water, human urine, gut, mouth, and skin [31]. However, the method assumes that all the observed microbial populations in the sink community come from source environments and ignores the fact that some active microorganisms can undergo rapid reproduction after entering the sink. When the abundance of immigrants increases, the source tracking method cannot fully explain this fraction of community composition from known sources. This limitation leads to the observation that sometimes the majority of the sink community is labeled as unknown, whereas only a small proportion can be explained by known sources. The observation of unknown sources is especially common in systems with high microbial activities, such as wastewater treatment processes [32–34].

The third approach uses the neutral model (Fig. 2c) developed by Sloan and coworkers [35]. By determining the abundance-frequency distribution of individual microbial species, a species-independent immigration probability *m* is calculated, which is uniform for every community member. A small *m* value suggests that the community as a whole is comprised of a low proportion of immigrants from the source. This model has been frequently used to evaluate the relative importance of neutral mechanism and directly assess the immigration rate at community level by calculating the *m* value. Using this model, Ayarza and Erijman revealed that neutral process was important in the assembly of activated sludge community [36]. In a drinking water distribution system, the model was used to demonstrate that the role of immigration from city water supply to tap water was higher at the proximal end than at the distal end of indoor plumbing [22]. Likewise, studies have compared the *m* values to reveal higher immigration impact in planktonic communities than in sedimentary communities in Yangtze River [37], and in deep-water communities than in surface water communities [38]. Despite the success of the model in explaining the general trend of abundance-frequency distribution, there are always species significantly deviating from the S-shape fitting curve. This is likely caused by assuming a constant immigration rate for all community members. It ignores the fact that immigrants with active microbial growth in the new environments can become more abundant than the prediction.

All the three methods described above merely enumerate the number of immigrants and cannot fully reveal the impact of microbial immigration on community functions. As microorganisms can carry considerably diverse activities after entering a new environment, in contrast to inert and homogeneous particles, it is important to address “how many microbial immigrants are able to actively contribute to ecological functions.”

## Quantifying immigration impact with the consideration of microbial activity

To quantitatively evaluate the immigration impact, both the abundance and activity of individual immigrants should be considered. Compared to determining abundance, assessing the activities or growth of individual immigrants in a given microbial ecosystem is challenging. In pure cultures, microbial activity can be assessed by measuring substrate consumption, metabolite production, or cell density change during a period of incubation. However, only a small fraction of microbes in nature can be cultivated and microbial activities determined in pure culture can differ drastically in a complex community under environmental conditions [39]. Sequencing 16S ribosomal RNA (rRNA) genes and other biomarkers is often used to identify microorganisms present in the environment but cannot effectively distinguish active populations from inactive or dormant species. Likewise, metagenomics reveals functional potentials of community members but cannot discern expressed and non-expressed pathways [40]. Directly sequencing rRNA can identify active microbes, but the consistency of this approach can be affected by the differences between rRNA content and microbial activity [41]. Sequencing messenger RNA (mRNA), i.e., metatranscriptomics, provides accurate identification of highly expressed genes and active populations from environmental samples [42], but this approach is challenged by the scarcity of well-annotated high-quality reference genomes [43]. Nucleotide sequencing can be coupled with methods such as microautoradiography [44], stable isotope probing [45], or nano secondary ion mass spectrometry [46] that label specific substrates to link substrates uptake activity with microbial identity. It is also possible to label and visualize specific active populations using fluorescence in situ hybridization designed to target rRNA [47]. These methods however cannot target all community members due to the cost and time associated with labeling individual substrates or organisms. In addition, metaproteomics and metabolomics can characterize the entire collection of proteins or metabolites of a given sample, and provide direct measurement of microbial activity, but also face challenges on preparing high-quality samples from complex environments and on linking proteins/metabolites with microbial identity [40]. Overall, it is still expensive and time-consuming using these ecological tools to quantify the in situ activities of most microbial populations in a complex ecosystem.

## Quantifying immigration impact at individual population level using ecogenomics-based mass balance approach

**Fig. 3:**
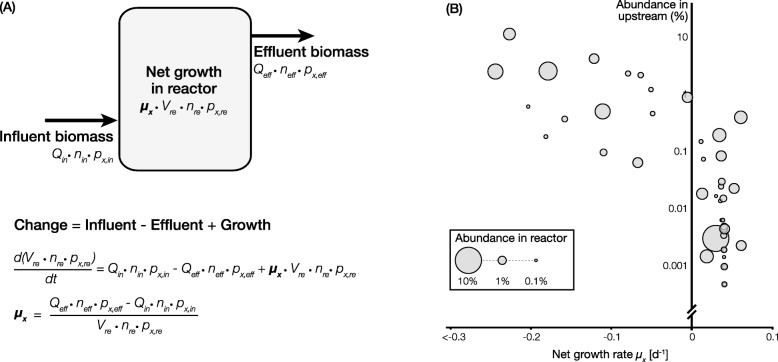
**a** Mass balance for a bioreactor with biomass input, output, and local growth. Notations in the model are described in the paragraph above. **b** An example of the net growth rate calculation result. *X*-axis denotes the net growth rate. The abundances of a community member in the upstream and downstream community are represented by the *Y*-axis and the size of the bubbles, respectively

Recent studies [48–51] have presented a high-throughput and quantitative way to assess the immigration impact on an open engineered water system by combining a mass balance concept with ecogenomics tools, i.e.*,* the ecogenomics-based mass balance. Using a biological reactor treating wastewater as an example (Fig. 3a), it receives influent biomass discharged from the upstream system with a flow rate *Q*_in_ and unit cell number *n*_in_. The relative abundance of any given microorganism *x* in the influent stream (*p*_*x*, in_) can be determined by 16S rRNA gene sequencing. Thus, the absolute number of *x* in the influent can be described as *Q*_in_ • *n*_in_ • *p*_*x*, in_ if *Q*_in_ and *n*_in_ are properly measured. Similarly, the number of *x* in the effluent can be described similarly as *Q*_eff_ • *n*_eff_ • *p*_*x*, eff_, and multiple influent/effluent streams are allowed. The growth of *x* in the reactor is expressed with a first order reaction (*μ*_*x*_ • *V*_re_ • *n*_re_ • *p*_*x*, re_), i.e., the product of a net growth rate constant *μ*_*x*_ and the absolute number of *x* in the reactor *V*_re_ • *n*_re_ • *p*_*x*, re_. When the reactor is operated at a steady state and the number of *x* in the reactor does not change through time, the sum of *x* in the influent, effluent, and its growth is zero, which allows the calculation of *μ*_*x*_. This growth rate constant is specific for each community member, which can be positive (i.e.*,* net growth and active ecological function) or negative (i.e.*,* net decay and minimal ecological function). When the growth rates of all community members are plotted against their abundance, one can easily tell whether the abundant populations in the upstream environment (major immigrants) are active or not in the downstream community. In the example illustrated in Fig. 3b, those located at the upper part of the figure tend to have negative growth rates, suggesting immigrants from the upstream exhibit low activity, although they still retain high abundance as indicated by the large bubble size. In contrast, active populations, those with positive growth rate, in the reactor are not derived from the upstream process. Overall, although immigrants represent a large proportion of observed abundance in this case, their contribution to the community function is likely low considering their negative growth rates.

Saunders et al. first developed this method and studied three WWTPs [48]. Thirty-five percent of the observed species in activated sludge reactors were also detected in the influent wastewater, suggesting a strong immigration impact. However, the mass balance revealed that majority of the shared species had negative net growth rate, suggesting they did not actively contribute to the metabolisms in activated sludge. There were a few immigrants with positive growth rate in activated sludge, indicating that they were indeed active in situ. Overall, the authors concluded a modest impact of immigration on the activated sludge community, considering there were both inactive and active immigrants with moderate abundance.

Mei et al. applied the ecogenomics-based mass balance approach to anaerobic digesters that receive massive biomass from upstream activated sludge [49]. Based on the result that populations with negative net growth rate accounted for 25% of total sequences in digesters, a strong immigration impact with the feed aerobic wasted sludge was concluded. Phylogenetic analysis confirmed that inactive populations were associated with aerobes or facultative anaerobes, whereas active populations were associated with obligate anaerobes. This study also reported the bias associated with the use of 16S rRNA-based relative abundance as an activity indicator in environments under high immigration impact. It is possible that some immigrants, which are the major populations in the previous environment, can still contain high copy number of rRNA after moving to a downstream environment. Thus, rRNA-based calculation can overestimate the relative abundance and contribution of these populations in the downstream community [49]. Such rationale was further used to multiple full-scale digesters around the world [52, 53], and the findings revealed that immigration from feed sludge to the digester communities was a ubiquitous phenomenon and the extent of contribution was also influenced by the operation conditions and pretreatments related to the anaerobic digestion.

The ecogenomics-based mass balance approach was also applied to an industrial WWTP to demonstrate its effectiveness in teasing apart the interaction between neutral and niche-based mechanisms [50]. In the studied WWTP, immigrants from an upstream anaerobic reactor were inactive (net growth rate ≤ 0) and represented a negligible fraction (1% of the total sequences) in the downstream activated sludge community, implying a weak immigration impact. But these immigrants were found to affect the prediction of key environmental parameters from community composition using a machine-learning tool [54]. To do so, a supervised learning regressor was first trained on a set of samples with known physiochemical parameters such as temperature, pH, and nutrient concentrations and then used to predict the target values of the remaining samples. Parameters with higher prediction accuracy played more important roles in shaping the microbial community. After removing inactive immigrants from the downstream community, the prediction accuracy greatly improved. This result suggests that more cautious interpretation should be made to identify the key environmental parameters based on community composition. Commonly used methods, including k-means clustering [55], principal components analysis [56], principal coordinate analysis [57], non-metric multidimensional scaling [58], and redundancy analysis [59], solely rely on DNA-based microbial abundance, but pay little attention to the existence of inactive immigrants. The efforts of correlating observed species abundance with environmental conditions would be notably biased in an open ecosystem where inactive populations are introduced by immigration.

Wastewater systems are often designed with complex process configuration where high biomass flux from one process to the next can take place. Sampling and controlling in these systems at different temporal and spatial scales are easier than natural environments. Therefore, these environments present an excellent opportunity to apply the ecogenomics-based mass balance approach to quantify the contribution of microbial immigration to community composition and function. Furthermore, this method can be applied to non-wastewater systems where microbial immigration is commonly present but the contribution is rarely quantified. The differentiation of inactive populations is specifically valuable in those environments where the growth rates of microorganisms can be more heterogeneous than in highly selective wastewater systems. For example, biofilm growth in the drinking water distribution pipe has been recognized as an important process that affects drinking water quality [21, 22]. The mass balance model can be used to characterize the growth and immigration of different organisms in the biofilm, especially those posing risks to human health. To do so, a section of pipe can be considered as the control volume, with fresh city water and tap water as the influent and effluent, respectively. Organisms that can scavenge substrates in the pipe will exhibit higher growth rates and can be released into the tap water. Different conditions and parameters related to the mass balance can be tested to assess their impacts on immigration. Some of them include the disinfection methods of the city water supply, the sizes and materials of the pipe, the temperature of the environment, and the period of the water stagnation. These results can provide guidance to improve drinking water quality and prevent waterborne disease outbreak from the aspect of microbial ecology. While the measurements of mass flux and cell count related to biofilm can be challenging, they can be solved for example by harvesting the biofilm after a period of development and by enumerating cell number with flow cytometry as demonstrated in a recent study [51]. Optical coherence tomography is another effective and non-destructive way to determine the biofilm mass and possibly mass change (e.g., in term of volume) on the pipe inner surface [60]. In other systems where diverse ecological functions are carried out, functional genes, such as *mcrA* for methanogenesis and *amoA* for nitrification, can be used to monitor a subset of populations with specific function(s) instead of 16S rRNA gene. Besides marker genes, metagenomics and metatranscriptomics can be used to estimate the abundance and activity, respectively, of individual immigrants with higher resolution. In addition, the immigration of viruses [61] and eukaryotes [62] from an upstream process to a downstream process can be monitored and quantified, in addition to prokaryotic populations.

The ecogenomics-based mass balance approach is effective in differentiating active and inactive populations resulted from immigration than the three methods discussed earlier that do not consider immigrants’ activities (Table 1). It evaluates immigration impacts by considering the abundance and activities of all immigrants. The net growth rate is calculated in a high-throughput manner and is specific for individual immigrants, unlike an index derived for a whole community from other three methods. The method can be further applied to complex ecosystems by including multiple influent and effluent streams in the calculation. Still, the mass balance approach should be used carefully with good experimental design. It assumes that the system is in a steady state and requires multiple measurements of cell number and biomass flux within a properly defined control volume. When 16S rRNA gene is used as the biomarker, biases associated with DNA extraction, PCR amplification, rRNA copy number, and amplicon sequencing are still present [63]. The calculated net growth rate is only a proxy of the in situ activity and cannot perfectly reflect the behaviors of individual community members. It remains challenging to apply the approach to systems with attached microbial growth such as granular sludge bioreactors, due to the difficulty in estimating total biomass and biomass flux, as well as the heterogeneity in microbial compositions within the consortia. Thus, more research efforts are needed in this direction.

**Table 1:** Comparison of the commonly used methods that quantify immigration impact and the ecogenomics-based mass balance

Method	Venn diagram	Source tracking	Neutral model	Ecogenomics-based mass balance
Abundance of total immigrants	✓	✓	✓	✓
Abundance of individual immigrants	X	X	X	✓
Activity of individual immigrants	X	X	X	✓
Multiple upstream environments	✓	✓	X	✓
Multiple downstream environments	✓	X	X	✓
Cell number estimation	X	X	X	✓
Flux measurement	X	X	X	✓
Steady-state assumption	X	X	X	✓
Repeated sampling	X	✓	✓	✓

## Conclusion

Microbial immigration is a ubiquitous and important process occurring in engineered water systems, and it allows microbes present in an upstream system to influence the microbial assembly and function in a downstream receiving system after entering. To understand the impact of microbial immigration, qualitative and quantitative methods are necessary and have been developed. Commonly used methods are recognized to have limitations in quantifying the immigration impacts. The ecogenomics-based mass balance approach provides a solution by quantitatively determining the activity profile of all microbial populations in a community. This approach can effectively identify inactive populations, especially those resulted from immigration, and pinpoint microorganisms that are actually carrying out the process of interest. Furthermore, when coupled with methods such as machine learning, it can better identify key environmental parameters affecting system performance, which can guide the monitoring and designing of biological processes. It is foreseen that such an approach can be widely applied to various engineered and possibly natural environments, where the contribution of microbial immigration remains to be further characterized.

## Data Availability

Not applicable.

## Abbreviations

mRNA: Messenger RNA
rRNA: Ribosomal RNA
WWTP: Wastewater treatment plant
